# Effects of G-Quadruplex Topology on Electronic Transfer Integrals

**DOI:** 10.3390/nano6100184

**Published:** 2016-10-15

**Authors:** Wenming Sun, Daniele Varsano, Rosa Di Felice

**Affiliations:** 1 China Building Materials Academy, Beijing 100024, China; swm@mail.sdu.edu.cn; 2 Center S3, CNR Institute of Nanoscience, Via Campi 213/A, Modena 41125, Italy; daniele.varsano@nano.cnr.it; 3 Department of Physics and Astronomy, Universsity of Southern California, Los Angeles, CA 90089, USA

**Keywords:** G-quadruplex, DNA, electronic coupling, transfer integrals, structure, density functional theory

## Abstract

G-quadruplex is a quadruple helical form of nucleic acids that can appear in guanine-rich parts of the genome. The basic unit is the G-tetrad, a planar assembly of four guanines connected by eight hydrogen bonds. Its rich topology and its possible relevance as a drug target for a number of diseases have stimulated several structural studies. The superior stiffness and electronic π-π overlap between consecutive G-tetrads suggest exploitation for nanotechnologies. Here we inspect the intimate link between the structure and the electronic properties, with focus on charge transfer parameters. We show that the electronic couplings between stacked G-tetrads strongly depend on the three-dimensional atomic structure. Furthermore, we reveal a remarkable correlation with the topology: a topology characterized by the absence of *syn*-*anti* G-G sequences can better support electronic charge transfer. On the other hand, there is no obvious correlation of the electronic coupling with usual descriptors of the helix shape. We establish a procedure to maximize the correlation with a global helix shape descriptor.

## 1. Introduction

Artificial G-quadruplexes engineered to assemble with four parallel G strands and no terminal loops are viable electrical conductors up to the scale of 100 nm, at odds with double-stranded DNA molecules of comparable length [1]. This remarkable evidence will boost exploitation of these molecules in nanotechnology [2,3]. In order to achieve control in this field, it is necessary to know how the electrons behave, and in particular how strongly electrons residing in adjacent G-tetrads are coupled to each other and can sustain electron or hole transfer.

Natural G-quadruplexes have long been known for their inherent biological relevance. Special G-rich nucleic acid sequences that occur in the telomeric region of chromosomes, as well as in regulatory and transcription regions, may fold into a quadruple motif [4]. These “monomolecular” (from a single parent strand) genetic G-quadruplexes are only 2–4 G-tetrad long and can fold with parallel or antiparallel strands. They may be responsible for chromosome stability. Recently, G-quadruplex folding has been observed in vivo in human cells, for both DNA and RNA [5,6].

G4-motifs are genetic sequences that can fold in a quadruple helix in certain circumstances, even during replication. G-quadruplexes folded from such motifs have been classified in terms of structural topologies, which are dictated by the succession of syn and anti glycosidic bond angles (GBA), as well as by the base sequence and loop length [7,8]. Note that the possible alternation of syn and anti GBA’s in the quadruplex develops upon folding. It was observed that the circular dichroism signal is a fingerprint of topology group [9,10]. This evidence raises the question whether the sequence, structure and folding topology can be exploited to tune the optical and electronic properties of G-quadruplexes. This achievement would be a landmark for exploitation of this versatile biological motif for therapeutical and nanotechnology applications. Thus, we investigated the relation between the quadruplex topology and the transfer integral indicator of the electronic structure, by means of density functional theory (DFT) calculations. This particular indicator will eventually determine whether the G-quadruplex can be used as an electrical conductor and as a target (e.g., for drugs) to control the propagation of oxidative damage in the human body by tuning the folding of G-rich sequences.

## 2. Results

### 2.1. Choice of Relevant G4 Structures, Spanning Viable Topologies

G-quadruplex structures can be described in terms of three topology groups [9]. Group I is constituted of quadruplex stems in which the four strands are parallel and the guanines are all *anti*. Group II is constituted of antiparallel quadruplexes in which successive guanines along a strand have the same (*anti*-*anti* or *syn*-*syn*) or distinct (*anti*-*syn* or *syn*-*anti*) GBA’s. Group III is constituted of antiparallel quadruplexes in which successive guanines along a strand have only distinct (*anti*-*syn* or *syn*-*anti*) GBA’s. Consistently with the choice adopted in optical experiments [9], we have selected structures available in the nucleic acid database, as reported in Table 1 and Table 2. For each structure, we have taken into account all the NMR models, for a total of 142 structural models. From each structural model, we have extracted all the possible intra-strand G-G couples for DFT transfer integral calculations, for a total of 960 of frozen G-G DFT calculations (248 for group I, 392 for group II and 320 for group III), which are a viable sample for statistical analysis.

### 2.2. Strength of Electronic Coupling

We find that the parallel topology boosts electronic charge transfer through G-quadruplexes. The strength of the electronic coupling between adjacent guanines is represented by the transfer integral V_IF_, which was computed through the Marcus-Hush two-state model as implemented in Gaussian09. Technical details are included in Section 4. For a clean data set, we computed only intra-strand transfer integrals. Against comparison with a higher level of theory [25], we find that the two-state model is a good approximation, at least in G-G stacked couples.

**Figure 1:**
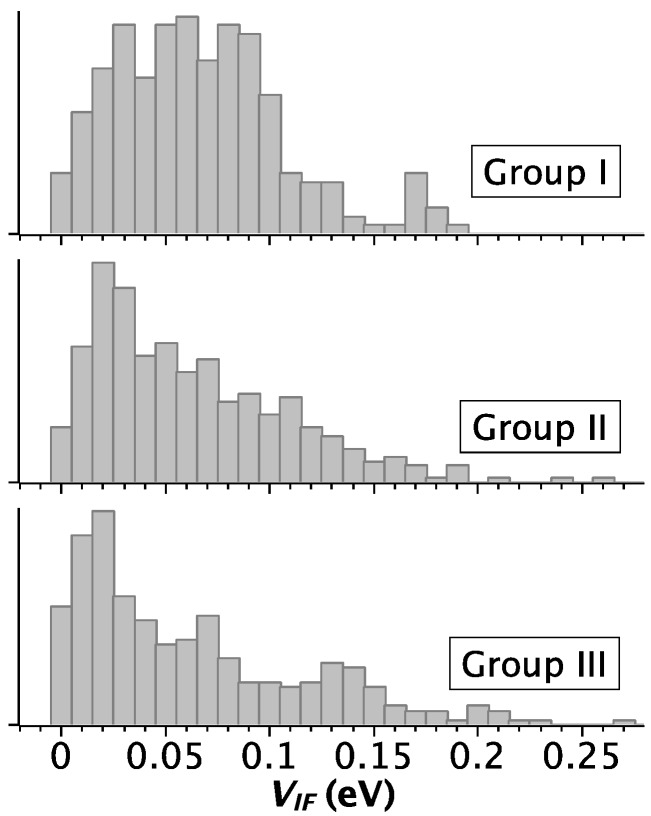
Distributions (normalized counts) of the computed transfer integral values for the three topology groups across the NMR structural models. The vertical axis reports the normalized counts. The group topologies are sketched in Figure 2 of Reference [9].

Figure 1 summarizes the statistical distribution of the transfer integral across all the G-G models, separately for the three topology groups. For group I we obtain an average transfer integral with standard deviation of 0.07 ± 0.04 eV. For both group II and group III the average values with standard deviations are 0.06 ± 0.06 eV. In all the cases the distribution is very broad, which was also observed in other studies of duplex and quadruplex DNA [25,26,27,28]. This finding confirms accumulating evidence that charge transfer through DNA should be analyzed taking into account the dynamical flexibility, in order to attain a quantitative description of realistic experimental conditions. Our results identify a special correlation between the strength of electronic coupling and the topology. Although the average values are almost identical in all groups, the pattern of the distributions in Figure 1 reveals that the average value is representative only for group I, where it is a good approximation of the most probable value, ~0.05 eV. In groups II and III, the distribution is not regular and the most probable value is ~0.01 eV, much smaller than that of group I (see also Supporting Figure S1). Thus, we can safely conclude that the group II and III topologies, with antiparallel strands and syn-*anti* and *anti*-*syn* GBA sequences, are detrimental to charge transfer through the quadruplex axis. These results are in line with experimental findings by Porath and coworkers, who showed that 4-stranded parallel G-quadruplexes exhibit a superior electrostatic polarizability and larger thickness than monomolecular G-quadruplexes when deposited onto mica [29]. Indeed, only 4-stranded parallel G-quadruplexes are able to transport electrical currents in such a solid-state configuration [1].

### 2.3. Correlation between Helix Shape Parameters and Electronic Coupling Parameters

We find that the quadruplex shape and the electronic parameters vary substantially across the NMR structures. Furthermore, None of the conventional helix shape parameters correlates significantly with indicators of the electronic structure.

**Figure 2:**
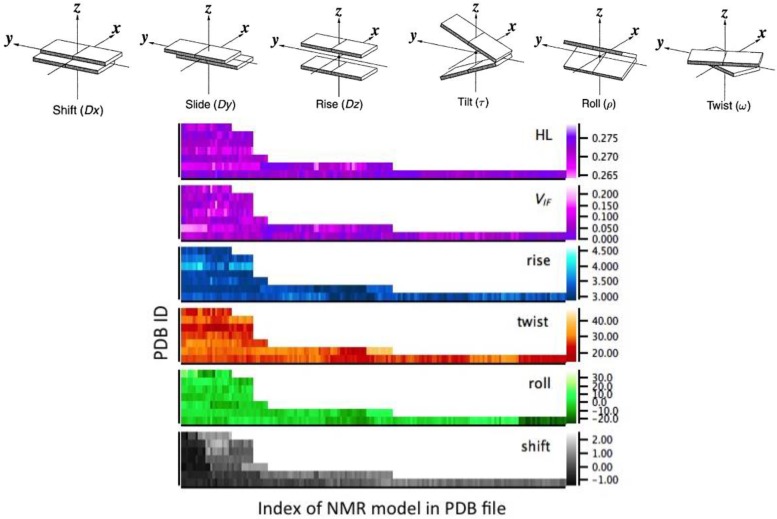
Top: illustration of the inter-base helix parameters between stacking guanines. Bottom: heat mapss of the structural and electronic parameters on which correlations have been evaluated. The 7 structures of group I and group II are arranged on the vertical axis, from bottom to top in each panel: PDB ID 1XAV, 2O3M, 2GKU, 2HY9, 2JPZ, 2KZD, 186D. The horizontal axis represents the different representative conformations (or NMR model) deposited for each structure in the nucleic acid database (details in Table 3). The scale is shown on the right vertical axis: HL and *V_IF_* are in eV, rise and shift are in Å, twist and roll are in degrees.

The quadruplex shape has been evaluated with the software Curves 5.3 [30]. This methodology implemented in this software applies only to regular Watson-Crick-like conformations, namely with the *anti*-*anti* uniform GBA sequence. Thus, for this analysis we considered only the structures in groups I and II (Table 1). Attention has been focused on the local inter-base intra-strand parameters that have been evaluated for stacked G-G couples (Figure 2, top). Moreover, for group II we have discarded all the parameters evaluated for *syn*-*syn*, *syn*-*anti* and *anti*-*syn* G-G couples (Table 3). Heat maps of two electronic parameters—*HL* and *V_IF_*—and four quadruplex shape parameters—rise, twist, roll and shift—are illustrated in the bottom part of Figure 2. HL is the energy gap between the highest occupied molecular orbital (HOMO) and lowest unoccupied molecular orbital (LUMO) of the +1 charged G-G couple, which is relevant to hole transfer. *V_IF_* is the computed transfer integral. The helix shape parameters are those illustrated in Figure 2. We can see big variations for all the parameters, distributed among all the structures. The roll of most conformations for all the structures is around the mid-range value. The twist of all conformations for structure 2JPZ is around the minimum value of 20 degrees; also for structure 186D the twist mostly assumes small values in all the 21 conformations of G-G couples. The other quantities are more uniformly spread. Considering all the 395 G-G couples together, the ranges are: *V_IF_* = 0.000 − 1.193 eV, *HL* = 0.264 − 0.276 eV, rise = 2.90 − 4.28 Å, twist = 14.27 − 41.37 degrees, roll = −25.58 − 26.76 degrees, shift = −1.43 − 1.81 Å.

**Figure 3:**
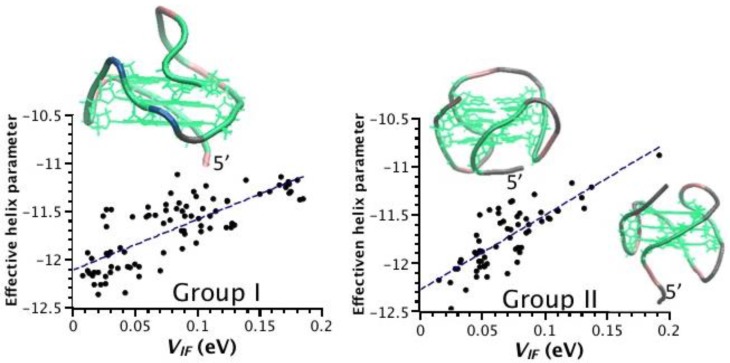
Scatter plots to illustrate the structural-electronic correlation for topology groups I and II. The insets illustrate the representative structures for group I (2O3M) and group II (2GKU top left, 2JPZ bottom right).

We have calculated the Pearson’s correlation coefficient [31] between *V_IF_* and each of the helix shape parameters shown in Figure 2—*P*(*V_IF_*-rise), *P*(*V_IF_*-twist), etc.—as well as between *V_IF_* and *HL*–*P*(*V_IF_*-*HL*). The *V_IF_*-HL Pearson’s correlation coefficients are reported in Table 1. We find a negative correlation between *V_IF_* and *HL*, which is consistent with the definition of the transfer integral in terms of the energy splitting at the reaction coordinate (Section 4). For the structures of group I and II the absolute value is larger than 0.7, with the exception of 1XAV; for the structures of group III smaller values occur. The *V_IF_*-helix Pearson’s correlation coefficients are reported in detail in the Supplementary Materials (Table S1). Here we discuss the salient features. The Pearson’s correlation coefficient does not reveal any remarkable correlation between *V_IF_* and the inter-guanine helix shape parameters: they are scattered between 0.07 and 0.69 in absolute values; some of them even vary between positive and negative correlation. Intuitively, one would think that the stacking distance (rise shape parameter) is a particularly crucial factor in determining the value of *V_IF_*: the larger the rise, the smaller the electronic coupling, with a negative correlation. However, we find a large spread of *P*(*V_IF_*-rise), from −0.63 to 0.17, with positive coefficients for structures with PDB ID’s 1XAV and 2HY9. Then we have searched for a homogeneous linear combination of the shape parameters that maximizes the Pearson’s correlation coefficient for each structure (Section 4.3). The values are much more meaningful, ranging between 0.49 to 0.85 for individual structures, while they are 0.59 and 0.44 for group I and II, respectively. The fact that cumulatively the correlation coefficients are smaller means that different linear combinations apply to different structures of the same group. Indeed, we note that in each group there are structures that conform to the intuition of a high negative correlation between *V_IF_* and rise and other structures that do not. We have thus looked more closely at the origin of the NMR studies, in order to make a selection. In group I we note that the quadruplex with PDB ID 2O3M [12] has a reasonable *P*(*V_IF_*-rise) value and that also *P*(*V_IF_*-twist) is negative, while these coefficients are oddly both positive in the quadruplex with PDB ID 1XAV [11]. Having these structures a parallel topology, one would expect a very regular behavior and similar to each other. They were resolved by different experimental groups, with PDB ID 1XAV being prior. Among the structures of group II, quadruplexes with PDB ID’s 2GKU [20] and 2JPZ [15] have the largest negative correlation coefficients of *V_IF_* with rise and twist: PDB ID 2GKU quadruplex [20] was resolved by the same group that solved PDB ID 2O3M; PDB ID 2JPZ quadruplex [22] was solved by the same group that solved PDB ID 1XAV. PDB ID 186D [17] is very old from 1994. PDB ID 2KZD quadruplex [16] was also resolved by Phan and coworkers (as PDB ID’s 2O3M and 2GKU), while PDB ID 2HY9 [14] was resolved by Yang and coworkers (as PDB ID’s 1XAV and 2JPZ). We have tried a restricted analysis of structure-function correlations using a limited set of structures resolved after 2006 and for which *P*(*V_IF_*-rise) is negative with large absolute value: We take PDB ID 2O3M as representative of group I, PDB ID’s 2GKU and 2JPZ as representative of group II. We find that the Pearson’s correlation coefficient between V_IF_ and the helix shape can be maximized by taking a unique homogeneous linear combination of the helix shape parameters. Specifically, the linear combination of the opposite of the rise, 1/2 of the roll and −5/3 of the twist maximizes the shape-*V_IF_* correlation. A slight further improvement is achieved by including also −1/3 of the shift. Scatter plots obtained for group I and group II using this combination of the helix shape parameters are shown in Figure 3 and reveal a significant linear correlation: the linear fits have a *R^2^* of 0.62 and 0.65 for group I and group II, respectively. For the restricted set of conformations represented in Figure 3, spanning parallel and hybrid topologies of the human telomeric sequence, the global shape variable can be tuned to design efficient molecular wires based on G-quadruplex.

## 3. Discussion

As a note of caution, we conclude by remarking that the representative models for NMR structure do not have a definite precision and collecting all of them in the statistical analysis may overweigh some features that hide the true shape-electronic correlations. On the other hand, Lech and collaborators reported that such correlations are hidden also in a molecular dynamics simulation. Moreover, our preliminary analysis of correlations between shape and transfer integral over regular snapshots from a 10 μs-long trajectory of a parallel quadruplex does not reveal a clear correlation. It may be necessary to filter the trajectory by eliminating internal distortions, which deserves full attention for a separate work.

In the present stage, we propose that, although no single shape parameter is responsible for the value of the electronic coupling, the inter-guanine parameters in the combination outlined above explain the electronic couplings in the experimental structures of groups I and II, characterized by different topologies, to a fair degree of confidence. While the value of the electronic coupling is highest for the topology of group I, the electronic-shape correlation is fairly independent of the topology.

## 4. Materials and Methods

### 4.1. Structural Analysis

The version Curves 5.3 that we have used to extract shape parameters supports the analysis of 4-stranded DNA structures. What really counts for the correct use of Curves is the sequence of glycosidic bond angles, which should be anti-anti as in B-DNA, because otherwise one should rotate locally the reference system to calculate shape parameters. This is true for inter-base parameters, which are the only parameters we are looking at. For other parameters, such as tip and opening, there may be issues; but not for those chosen by us, which we believe are the most relevant for charge transfer. This is the reason why the group of structures for the analysis of correlations is restricted to those in the rightmost column of Table 4.

### 4.2. Computational Approach—Electronic Structure Calculations

**Figure 4:**
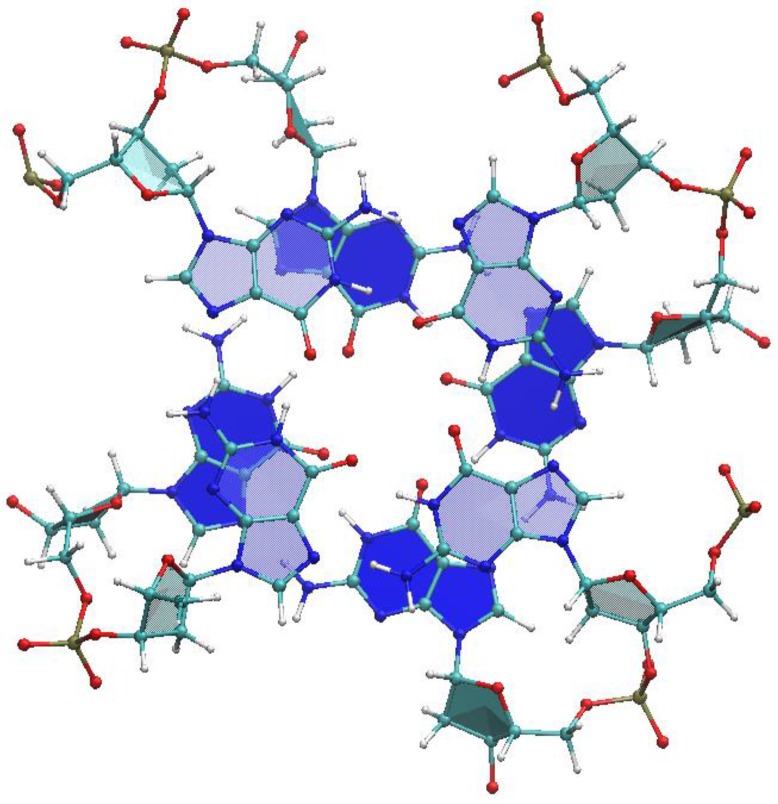
Exemplifying structure of a (G4)_2_ dimer. This is taken from the G-quadruplex 1XAV, which contains three tetrads. The tetrads shown here included guanines 4, 8, 13, 17, 5, 9, 14, 18, as labeled in the PDB file.

From each G-quadruplex we extracted one, two or three dimers of stacked G-tetrads, according to Table 2. A dimer is the fragment of the quadruplex constituted of two adjacent tetrads (Figure 4) and we label it (G4)_2_. These fragments, deprived of the backbone, were subjected to quantum mechanical (QM) calculations in the framework of density functional theory.

DFT calculations were performed with Gaussian09 using the Becke Half-and-Half (BHH) functional [32] and the 6-31+G* basis set. The BHH functional comprises ½ Hartree-Fock exchange, ½ Slater exchange and ½ PW91-LDA correlation. Being derived from the rigorous adiabatic connection formula for the exchange-correlation energy of Kohn-Sham DFT [33,34,35,36], the BHH functional rests on a clear theoretical basis, rather than on an empirical choice for the amount of exact exchange. For different π-stacked aromatic complexes, this density functional gave results in agreement with post-HF calculations and experimental data [37,38,39,40].

The aqueous environment was simulated in the framework of the polarizable continuum model [41].

The sugar-phosphate backbone has been omitted from these calculations and the dangling bond was saturated with a H atom. This choice was based on previous evidence that frontier orbitals, which are mostly responsible for electronic coupling through π-stacked nucleotides, are essentially localized on the base hetero-rings [42,43]. We are thus representing a framework in which any influence of the backbone topology on the electronic structure and charge transfer is indirect. Namely, the backbone moieties do not participate in charge transfer but impose structural conformations that are more or less conducive to charge transfer along the helix axis. The backbone effect on electronic couplings in DNA has been widely discussed by several authors and is discussed in reviews [44]. In particular, Beratan and coworkers revealed that base interactions clearly dominate the bridge-mediated coupling interactions, even in the presence of the bridging backbone [45].

The strength of the electronic coupling between adjacent molecules is represented by the transfer integral, which was computed through the energy-splitting in dimer model as implemented in Gaussian09. The transfer integral crucially depends on tiny atomic movements that change the shape of frontier electron orbitals, specifically the highest occupied molecular orbital (HOMO) and the orbital lying immediately below in the energy scale (HOMO-1) of the neutral complex. The transfer integral of a selected (G4)_2_ dimer is related to the energy splitting between HOMO and HOMO-1 of the dimer, which in turn are determined by the HOMO of the unit guanine. Thus, we computed intra-strand and inter-strand transfer integrals between guanines in consecutive tetrads. When two guanine molecules approach each other, their interaction induces an energy splitting between the two highest occupied molecular orbitals in the dimer. The transfer integral V_IF_ can be expressed in terms of the energy difference between the HOMO and HOMO-1 in the interacting guanine dimer [46,47]: $V_{IF}=\sqrt{{\left(E_{HOMO}-E_{HOMO-1}\right)}^{2}-{\left(\varepsilon_{1}-\varepsilon_{2}\right)}^{2}}$, where $\varepsilon_{1}$ and $\varepsilon_{2}$ are the energy levels of the HOMOs of the two interacting guanines. If the two interacting guanines were identical we would have $\varepsilon_{1}=\varepsilon_{2}$. In our work, however, the geometries of two stacked guanines are never identical: they are not ideal structure formulas but come from real NMR data and have not been further optimized. Consequently, $\varepsilon_{1}\neq \varepsilon_{2}$ in the formula for $V_{IF}$.

We are aware that the two-state model for *V_IF_* introduces errors in the estimated quantity, which can in principle be bypassed by more accurate theories [25,37,40]. We chose in this work a simple method that can routinely be applied to several fairly large structures, which are computationally prohibitive for more sophisticated approaches. We verified that this method gives satisfactory accuracy for the transfer integral of two stacked guanines. We used the standard G-G stacking geometry as an example: the transfer integral computed by us with the energy splitting approach and the technical ingredients specified above is 0.083 eV. This is a good approximation of the value 0.075 eV determined by Migliore and co-workers using a refined formula for $V_{IF}$, a more complete basis set for the electronic wave functions and diabatic states for the dimer [25]. To our purposes of analyzing the topological dependence of $V_{IF}$ and revealing possible structure-$V_{IF}$ correlations this accuracy is sufficient and allows us to sample about a thousand relevant geometries. Furthermore, the similar fragment charge difference approach was successfully employed for the investigation of several G-quadruplex conformations [26]: the reliability of this and other semi-empirical methods is also discussed in the existing literature [47,48,49].

### 4.3. Statistical Analysis—Maximizing the Pearson’s Correlation Coefficient with the Transfer Integral for a Linear Combination of Helix Shape Parameters

Let us define the variables

(1) xn=shift, slide, rise, d1sintilt, d2sinroll,12d1(sintwist+costwist) andz=∑n=16αnxn,

with n ranging from 1 to 6, *d_1_* = 7.4 Å and *d_2_* = 4.0 Å being the size of the long and short side of guanine, respectively. *z* is a homogeneous linear combination of the local inter-base helix parameters, with coefficients $\alpha_{n}$ that we wish to optimize to maximize the Pearson’s correlation coefficient between *z* and the transfer integral V*_IF_*–*P(z,V_IF_)*, or between *z* and the HOMO-LUMO gap *HL*–*p(z,HL)*.

For *x_n_*, *z*, *V_IF_* and *HL* we have several instances, because we have considered different NMR structures, each of them with a number of models. So we use another index *i* that labels the G-G stacked couples, as a superscript. *N* is the total number of such couples in each considered group. A group could be Group I, Group II or Group III, or any single pdb (e.g., 2O3M), or any other classification that we want to consider.

By definition, the Pearson’s correlation coefficient between our target structural variable z and a generic electronic variable *t* (*t* is either *V_IF_* or *HL*) is:

(2) $$P\left(z,t\right)=\frac{\sum_{i=1}^{N}\left(z^{i}-\bar{z}\right)\left(t^{i}-\bar{t}\right)}{\sqrt{\sum_{i=1}^{N}{\left(z^{i}-\bar{z}\right)}^{2}}\sqrt{\sum_{i=1}^{N}{\left(t^{i}-\bar{t}\right)}^{2}}}$$

Now let us replace *z_i_* with its definition from Equation (1):

(3) $$P\left(z,t\right)=\frac{\sum_{i=1}^{N}\sum_{n=1}^{6}\alpha_{n}\left(x_{n}^{i}-{\bar{x}}_{n}\right)\left(t^{i}-\bar{t}\right)}{T\sqrt{\sum_{i=1}^{N}\sum_{n,m=1}^{6}\alpha_{n}\alpha_{m}\left(x_{n}^{i}-{\bar{x}}_{n}\right)\left(x_{m}^{i}-{\bar{x}}_{m}\right)}}$$

In Equation (3), $T=\sqrt{\sum_{i=1}^{N}{\left(t^{i}-\bar{t}\right)}^{2}}$ is a constant factor that can be excluded from the maximization problem.

Exploiting the definition of covariance, the problem of maximizing *P(z,t)* in Equation (3) can be recast as:

(4) $$Max\left(\frac{\sum_{n=1}^{6}\alpha_{n}cov\left(x_{n},t\right)}{\sum_{n,m=1}^{6}\alpha_{n}\alpha_{m}cov\left(x_{n},x_{m}\right)}\right)$$

The quantity $cov\left(x_{n},t\right)$ is a 6-component vector (for *n* = 1,…,6), say $\vec{b}$. The numerator in Equation (4) is thus the scalar product $\vec{\alpha }\cdot \vec{b}$, with $\vec{\alpha }$ being the 6-component vector formed by the coefficients $\alpha_{n}$. $A_{nm}=cov\left(x_{n},x_{m}\right)$ are the matrix elements of the 6×6 matrix A. In matrix-vector notation, Equation (4) becomes:

(5) $$Max\left(\frac{\vec{\alpha }\cdot \vec{b}}{\sqrt{{\vec{\alpha }}^{T}A\vec{\alpha }}}\right)$$

Matrix diagonalization of A yields the eigenvalues $\lambda_{n}$ and eigenvectors ${\vec{\delta }}_{n}$ (*n* = 1,…,6). By definition, the matrix A can be expressed in terms of its eigenvalues and eigenvectors:

(6) $$A=\overset{6}{\underset{n=1}{\sum }}\lambda_{n}{\vec{\delta }}_{n}{\vec{\delta }}_{n}^{T}$$

The 6-component vector $\vec{\alpha }$ can be expressed on the basis of the eigenvectors ${\vec{\delta }}_{n}$ with proper coefficients $a_{n}$:

(7) $$\vec{\alpha }=\overset{6}{\underset{n=1}{\sum }}a_{n}{\vec{\delta }}_{n}$$

With these expression Equation (5) can be further developed to:

(8) $$Max\left(\frac{\sum_{n=1}^{6}a_{n}{\vec{\delta }}_{n}\cdot \vec{b}}{\sum_{n=1}^{6}a_{n}^{2}\lambda_{n}}\right)$$

For further manipulation to a manageable analytical solution, after defining and substituting $\tilde{a}=a_{n}\sqrt{\lambda_{n}}$ one obtains:

(9) $$Max\left(\frac{\sum_{n=1}^{6}{\tilde{a}}_{n}\left(\frac{{\vec{\delta }}_{n}\cdot \vec{b}}{\sqrt{\lambda_{n}}}\right)}{\sum_{n=1}^{6}{\tilde{a}}_{n}^{2}}\right)$$

Or

(10) $$Max\left(\frac{\sum_{n=1}^{6}{\tilde{a}}_{n}d_{n}}{\sum_{n=1}^{6}{\tilde{a}}_{n}^{2}}\right),\;d_{n}=\frac{{\vec{\delta }}_{n}\cdot \vec{b}}{\sqrt{\lambda_{n}}}$$

The expression in Equation (8) is maximum for ${\tilde{a}}_{n}=\frac{d_{n}}{\sqrt{\sum_{k=1}^{6}d_{k}^{2}}}$, and going backwards it is possible to write $a_{n}$ and then $\vec{\alpha }$, whose 6 components are the coefficients of the linear combination in Equation (1), namely the solution of the problem. From the diagonalization of the matrix *A* one thus obtains the coefficients that maximize the Pearson’s correlation coefficient P(z,t).

In summary, the steps of this procedure, which has been applied to obtain the data in the rightmost column of Table S1 (Supplementary Materials) and the scatter plots of Figure 3, are:

- compute the covariance of each inter-base helix parameter *x_n_* with *V_IF_* and with all the other inter-base helix parameters *x_m_*; the former is vector $\vec{b}$ (this vector can also be obtained for the electronic quantity *t* = *HL*), the latter is matrix *A*;
- diagonalize matrix *A*;
- use the eigenvalues and eigenvectors of *A* to determine the coefficients $a_{n}$ that maximize the shape-electronic Pearson’s correlation coefficient;
- calculate the Pearson’s correlation coefficient of this combination, which is an effective helix parameter, with *V_IF_* (and *HL*), reported in Table S1.

The scatter plots in Figure 3 have been obtained using a similar linear combination, but with the coefficients tuned to represent all the representative structures of groups I and II, with a reduced number of parameters (as discussed in Section 2.3). Such a global homogeneous structural quantity takes into account how the relevant part (inter-base parameters) of the entire helical shape correlates with the ability of the guanine stack to sustain charge transfer. It means that the global shape, rather a single parameter, is relevant for physicochemical function, at least for the specific function of charge transfer.

## 5. Conclusions

We have carried out density functional theory calculations of transfer integrals for G-G stacked couples extracted from G-quadruplexes of different topologies, and looked for correlations between this electronic quantities and helix shape parameters. Our results indicate that the parallel topology is characterized by the highest transfer integrals, relative to antiparallel and hybrid topologies. Furthermore, we find that it is not a single shape parameters that correlates with the electronic coupling, but rather a linear combination of inter-base parameters that embodies the global helix shape. This global shape parameter can be tuned to optimize charge transport through G-quadruplexes.

## Figures and Tables

**Table 1:** Summary of the computed structures and electronic correlation.

Group	PDB Code	Nr. G4 ^§^	*P*(*V_IF_*-*HL*) ^†^
I	1XAV	3	−0.49
	2O3M	3	−0.73
II	2GKU	3	−0.71
	2HY9	3	−0.87
	2JPZ	3	−0.81
	2KZD	3	−0.82
	186D	3	−0.78
III	2KOW	2	−0.92
	201D	4	−0.60
	143D	3	−0.57
	2KKA	2	−0.41
	2KM3	2	−0.94
	148D	2	−0.87
	2KF8	2	−0.94

^§^ Nr. G4 is the number of G-tetrads. ^†^ *P(V_IF_-HL)* is the Pearson’s correlation coefficient between the electronic structure parameters *V_IF_* (transfer integral) and *HL* (energy gap), which are defined and commented later.

**Table 2:** Summary of the computed structures.

	PDB Code	Nr. Models ^†^	Nr. Tetrads ^‡^	Nr. Guanines	Strand Directions	Parent Sequence
Group I	1XAV [11]	20	3	22	++++	5’-TGAGGGTGGGTAGGGTGGGTAA-3’
	2O3M [12]	11	3	22	++++	5’-AGGGAGGGCGCTGGGAGGAGGG-3’
Group II	2GKU [13]	12	3	24	++−+	5’-TTGGGTTAGGGTTAGGGTTAGGGA-3’
	2HY9 [14]	10	3	26	++−+	5’-AAAGGGTTAGGGTTAGGGTTAGGGAA-3’
	2JPZ [15]	10	3	26	+−++	5’-TTAGGGTTAGGGTTAGGGTTAGGGTT-3’
	2KZD [16]	10	3	20	+−++	5’-AGGGIAGGGGCTGGGAGGGC-3’
	186D [17]	7	3	24	+−++	5’-TTGGGGTTGGGGTTGGGGTTGGGG-3’
Group III	2KOW [18]	10	2	20	+−+−	5’-TAGGGTAGGGTAGGGTAIGG-3’
	201D [19]	6	4	28	+−+−	5’-GGGGTTTTGGGGTTTTGGGGTTTTGGGG-3’
	143D [20]	6	3	22	+−+−	5’-AGGGTTAGGGTTAGGGTTAGGG-3’
	2KKA [21]	8	2	23	+−+−	5’-AGGGTTAGGGTTAIGGTTAGGGT-3’
	2KM3 [22]	10	2	22	+−+−	5’-AGGGCTAGGGCTAGGGCTAGGG-3’
	148D [23]	12	2	15	+−+−	5’-GGTTGGTGTGGTTGG-3’
	2KF8 [24]	10	2	22	+−+−	5’-GGGTTAGGGTTAGGGTTAGGGT-3’

^†^ The number of models in the NMR structure from the protein databank. ^‡^ Number of G-tetrads that compose the G-quadruplex. Strand directions in the quadruplex stem: + means parallel, - means antiparallel. The first strand is + starting from the 5’ end of the parent strand. The parent sequence indicates the DNA G4-motif.

**Table 3:** Number of G-G couples from the G-quadruplex for the analysis in Figure 1, Figure 2 and Figure 3.

	PDB Code	Nr. G-G Couples for *V_IF_* * Statistics (Figure 1)	Nr. G-G Couples for Conformation Fluctuations and Structure-Electronic Correlations (Figure 2 and Figure 3)
Group I	1XAV	160	160
	2O3M	88	88
	total group I	248	248
Group II	2GKU	96	36
	2HY9	80	30
	2JPZ	80	30
	2KZD	80	30
	186D	56	21
	total group II	392	147
Group III	2KOW	40	0
	201D	72	0
	143D	48	0
	2KKA	32	0
	2KM3	40	0
	148D	48	0
	2KF8	40	0
	total group III	320	0

* *V_IF_* is the transfer integral, computed according to the details described in the Computational approach below.

**Table 4:** GBA sequence in the selected structures.

	PDB Code	Strand 1	Strand 2	Strand 3	Strand 4	Strand Directions
Group I	1XAV	4(a) 5(a) 6(a)	8(a) 9(a) 10(a)	13(a) 14(a) 15(a)	17(a) 18(a) 19(a)	++++
	2O3M	2(a) 3(a) 4(a)	6(a) 7(a) 8(a)	13(a) 14(a) 15(a)	10(a) 21(a) 22(a)	++++
Group II	2GKU	3(s) 4(a) 5(a)	9(s) 10(a) 11(a)	17(a) 16(s) 15(s)	21(s) 22(a) 23(a)	++−+
	2HY9	4(s) 5(a) 6(a)	10(s) 11(a) 12(a)	18(a) 17(s) 16(s)	22(s) 23(a) 24(a)	++−+
	2JPZ	4(s) 5(a) 6(a)	12(a) 11(s) 10(s)	16(s) 17(a) 18(a)	22(s) 23(a) 24(a)	+−++
	2KZD	2(s) 3(a) 4(a)	10(a) 9(s) 8(s)	13(s) 14(a) 15(a)	17(s) 18(a) 19(a)	+−++
	186D	3(s) 4(a) 5(a)	12(a) 11(s) 10(s)	16(s) 17(a) 18(a)	21(s) 22(a) 23(a)	+−++
Group III	2KOW	3(s) 4(a)	9(a) 8(s)	14(s) 15(a)	19(a) 20(s)	+−+−
	201D	1(s) 2(a) 3(s) 4(a)	12(a) 11(s) 10(a) 9(s)	17(s) 18(a) 19(s) 20(a)	28(a) 27(s) 26(a) 25(s)	+−+−
	143D	2(a) 3(s) 4(a)	10(s) 9(a) 8(s)	14(a) 15(s) 16(a)	22(s) 21(a) 20(s)	+−+−
	2KKA	2(s) 3(a)	9(a) 8(s)	15(s) 16(a)	21(a) 20(s)	+−+−
	2KM3	3(s) 4(a)	10(a) 9(s)	15(s) 16(a)	22(a) 21(s)	+−+−
	148D	1(s) 2(a)	6(a) 5(s)	10(s) 11(a)	15(a) 14(s)	+−+−
	2KF8	1(s) 2(a)	8(a) 7(s)	14(s) 15(a)	20(a) 19(s)	+−+−

The numbers indicate the guanine (DG) labels in the NMR structure file with the specified pdb code. In parentheses: a = *anti*, s = *syn*. The column “strand directions” is repeated from Table 1.

## Supplementary Materials

The following are available online at www.mdpi.com/2079-4991/6/10/184/s1. Table S1: Pearson’s correlation coefficients [31] between structural and electronic parameters. Figure S1: Gaussian fits of statistical distributions.

## Abbreviations

The following abbreviations are used in this manuscript:

GBA: glycosydic bond angle
DFT: density functional theory
Nr.: number
HOMO: highest occupied molecular orbital
LUMO: lowest unoccupied molecular orbital
*HL*: HOMO-LUMO gap
BHH: Becke half-and-half functional
NMR: nuclear magnetic resonance
PDB: protein data bank
