# Supplementary material for: Effects of G-Quadruplex Topology on Electronic Transfer Integrals
Source: Nanomaterials (Basel). 2016 Oct 15;6(10):184. doi: 10.3390/nano6100184 (PMC5245196; doi:10.3390/nano6100184)
Supplement: Supplementary file 1 [file nanomaterials-06-00184-s001.pdf]

Supplementary Materials: Effects of G-Quadruplex Topology on Electronic Transfer Integrals

Wenming Sun, Daniele Varsano and Rosa Di Felice

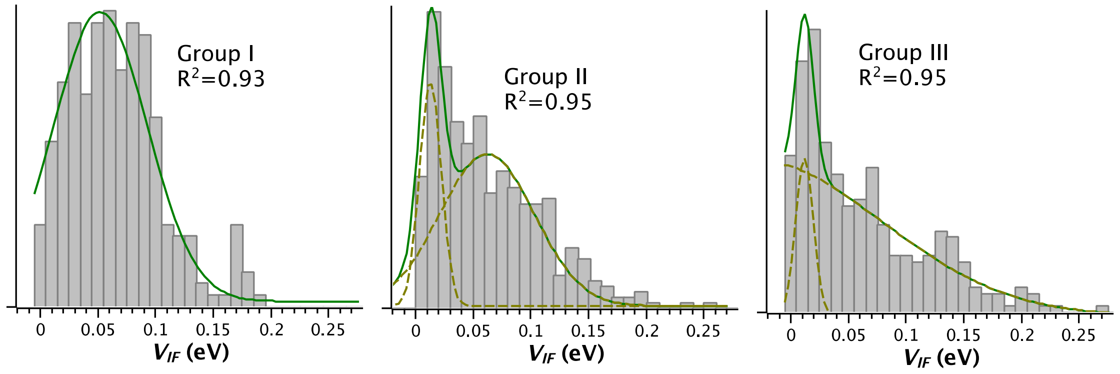

Figure S1. Single and multiple Gaussian fits of the distribution of transfer integral across the investigated NMR structures for group I, II and III from left to right. Although the distributions can in principle be fitted with other statistical models, Gaussians give a good agreement with the histograms, in terms of R^2. The distribution of group I can be fitted with a single Gaussian centered at 0.05 eV, with $R^2 = 0.93$. The distributions of group II and group III need a 2-Gaussian fit with the most intense peak at 0.01 eV and $R^2 = 0.95$. Group II, which has *anti-anti*, *syn-syn* and *syn-anti* (*anti-syn*) GBA sequences, also has a second peak at about 0.065 eV. In group III, which has only *syn-anti* (*anti-syn*) sequences, the second peak is unphysical.

Table S1. Pearson's correlation coefficients [1] between structural and electronic parameters §.

			Shift	Slide	Rise	Tilt	Roll	Twist	Max$_h$ $
V_{IF}	Group I (248)	1XAV	−0.087	−0.136	0.072	0.180	0.433	0.164	0.487
		2O3M	−0.690	−0.619	−0.501	−0.272	0.100	−0.246	0.839
		Group I	−0.368	−0.345	−0.249	0.058	0.305	0.028	0.594
	Group II (147)	2GKU	0.275	0.242	−0.559	0.510	0.457	−0.248	0.851
		2HY9	−0.025	−0.212	0.166	−0.383	0.032	0.357	0.670
		2JPZ	0.502	0.374	−0.632	−0.227	−0.174	−0.512	0.788
		2KZD	−0.230	−0.125	−0.185	−0.174	−0.295	0.263	0.622
		186D	0.420	0.346	−0.141	−0.079	−0.386	0.260	0.560
		Group II	0.176	0.098	−0.372	−0.046	−0.070	−0.109	0.437
HL	Group I (248) †	1XAV	0.176	−0.009	0.013	−0.059	−0.235	0.018	0.531
		2O3M	0.638	0.381	0.762	0.547	0.238	0.520	0.896
		Group I	0.345	0.158	0.468	0.062	−0.097	0.074	0.661
	Group II (147)	2GKU	−0.433	−0.596	0.398	0.186	0.113	0.163	0.858
		2HY9	−0.213	−0.143	0.342	−0.003	−0.234	−0.109	0.602
		2JPZ	−0.385	−0.137	0.741	0.322	0.464	0.564	0.804
		2KZD	−0.134	−0.212	0.514	−0.091	0.191	−0.103	0.732
		186D	0.029	−0.340	0.023	−0.558	0.213	−0.144	0.808
		Group II	−0.079	−0.204	0.405	−0.038	0.159	0.146	0.572

§ Representative structures for group I and group II are highlighted in yellow. † The number of contributing G-G stacked couples is indicated in parentheses. $ Max$_h$ is the linear combination of helix shape parameters that maximizes correlation between the structure and the electronic transfer integral in G-G pairs.

Reference

1. Pearson, K. Mathematical Contributions to the Theory of Evolution. III. Regression, Heredity and Panmixia. *Philos. Trans. R. Soc. London* **1896**, *187*, 253–318.
